# Supplementary material for: Content-rich biological network constructed by mining PubMed abstracts
Source: BMC Bioinformatics. 2004 Oct 8;5:147. doi: 10.1186/1471-2105-5-147 (PMC528731; doi:10.1186/1471-2105-5-147)
Supplement: Additional File 2 — The original results of the above study (non-essential files are deleted to keep the file size under the limit set by BMC bioinformatics). [file 1471-2105-5-147-S2.bz2 › chilibotAdditionalFile2/dip40/429ID10910894E10359/html/CCR1_CCL4.html]

 


 **CCR1** and **CCL4** 
  
Found 39 abstracts in PubMed, retrieved 39.  
 

 What does Google say? 
 PDF only 
| .edu only 

---

**Interactive relationship** (e.g. stimulation, inhibition, etc)

**Neutral relationship**- However, the remaining expression of  **CCR1**  and CCR5 correlated reciprocally with an ongoing unresponsiveness to the  **CCR1**  and CCR5 agonists CCL3 macrophage inflammatory protein 1alpha MIP 1alpha,  **CCL4**  MIP 1beta, and CCL5 regulated on activation, normal T expressed and secreted RANTES, all chemokines binding to these two receptors.  Ref: 12885942 J Leukoc Biol, 2003
- The incubation of lambda DNA in the reaction system of alloxan plus NADPH cytochrome P450 reductase  [ **CCR1** ]  fp2 in the presence of ferritin caused strand breaks after a lag  [ **CCL4** ]  time of about 5 min.  Ref: 7742795 Biol Pharm Bull, 1995
- COS cells transiently transfected with the murine homologue of human CCR 1  [ **CCR1** ]  bind murine MIP 1 alpha and human RANTES with Kds of 3.4 nM and 4.2 nM and murine MIP 1 beta  [ **CCL4** ]  with an EC50 of 8.9 nM.  Ref: 7594543 J Immunol, 1995
- The CC chemokine receptor CCR  [ **CCR1** ]  8 belongs to the seven transmembrane spanning receptor family and functionally responds to the eukaryotic CC chemokines I 309, thymus and activation regulated chemokine, macrophage inflammatory protein 1 beta  [ **CCL4** ]  MIP 1b and to the products viral MIP I and viral MIP II of the Kaposi associated herpesvirus HHV 8.  Ref: 10611408 Forum Genova < MedlineTA>Forum Genova , 1995

**Non-interactive relationship** (e.g. studied together, co-existance, homology, etc.)

- Moreover, the forskolin mediated increases in intracellular cAMP concentrations were inhibited by the ligands for  **CCR1** , CCL3,  **CCL4** , and CCL5, suggesting that the expressed  **CCR1**  was functional.  Ref: 12651617 Am J Pathol, 2003
- In the response to glucose, there was approximately 30 min lag  [ **CCL4** ]  time between the peaks of IRI and CPR  [ **CCR1** ]  in the normal controls and the patients with various diseases.  Ref: 6761899 Tohoku J Exp Med, 1982
- The most active chemokines for resting Th IL 10, in terms of calcium mobilization and in vitro migration, were in order of potency CCL2 monocyte chemoattractant protein 1, CCR2 ligand,  **CCL4**  macrophage inflammatory protein 1beta, CCR5 ligand, CCL3 macrophage inflammatory protein 1alpha,  **CCR1**  5 ligand, CCL17 thymus and activation regulated chemokine, CCR4 ligand, CCL1 I 309, CCR8 ligand, CXCL12 stromal derived factor 1, CXCR4, and CCL11 eotaxin, CCR3 ligand.  Ref: 11145678 J Immunol, 2001
- In analysis of responses of plasma IRI and CPR  [ **CCR1** ]  to tolbutamide or glucagon, there was a lag  [ **CCL4** ]  time longer than 10 min in the normal subjects.  Ref: 6761899 Tohoku J Exp Med, 1982
- However, MIP 1 beta  [ **CCL4** ]  was also an agonist for CC CKR3 but not CC CKR1  [ **CCR1** ] .  Ref: 7622448 J Biol Chem, 1995
- Furthermore, a longer lag  [ **CCL4** ]  time between the peaks of IRI and CPR  [ **CCR1** ]  in response to tolbutamide or glucagon did not necessarily indicate a simultaneous release of insulin and C peptide from the B cell, but a delayed release of the latter.  Ref: 6761899 Tohoku J Exp Med, 1982
- After 2 to 4 weeks, L. amazonensis infected mice had significantly delayed and depressed expression of inflammatory cytokines interleukin 12 IL 12, gamma interferon, IL 1alpha, IL 1beta, CC chemokines CC chemokine ligand 3 CCL3 macrophage inflammatory protein 1alpha MIP 1alpha,  **CCL4**  MIP 1beta, CCL5 RANTES, MIP 2, and chemokine receptors  **CCR1** , CCR2, CCR5 in foot tissues and draining lymph nodes compared to the expression in L. major infected controls.  Ref: 12874303 Infect Immun, 2003
- Characterization of the chemokine receptor specificity of MIP 1 beta  [ **CCL4** ]  3 69 showed that the truncated protein not only shares the ability of intact MIP 1 beta  [ **CCL4** ]  to induce Ca 2 signaling through CCR5, but unlike the full length protein, it also triggers a Ca 2 response via  **CCR1**  and CCR2b.  Ref: 12070155 J Biol Chem, 2002
